# Psychosocial challenges of children with disabilities in Sekhukhune District, Limpopo province of South Africa: Towards a responsive integrated disability strategy

**DOI:** 10.4102/ajod.v11i0.799

**Published:** 2022-07-28

**Authors:** Matthews M. Makwela, Elizabeth I. Smit

**Affiliations:** 1 Department of Social Work, Faculty of Health Sciences, North-West University, Mmabatho, South Africa

**Keywords:** psychosocial challenges, disability, children with disabilities, Limpopo, South Africa

## Abstract

**Background:**

Disability, and everything it encompasses, presents major challenges to individuals, families and communities worldwide. Children with disabilities (CWD) are marginalised and excluded in most societies. Discrimination and prejudice towards CWD are compounded by poverty, lack of essential services and support and sometimes a hostile and inaccessible environment.

**Objectives:**

The study sought to examine the psychosocial challenges experienced by CWD in the Sekhukhune district of Limpopo province, South Africa. Based on the identified, articulated and expressed challenges, the study sought to recommend improvement of the existing Integrated National Disability Strategy (INDS) for greater responsiveness to the needs of CWD at both provincial and local levels.

**Method:**

The interpretivist qualitative mode of enquiry was the chosen methodology for this study. Phenomenology and descriptive research designs guided the study. Purposive sampling was employed, and data were collected from 36 participants using three triangulated methods: individual in-depth interviews, focus group discussions and key informant interviews. Thematic data analysis was used to analyse data.

**Results:**

The findings revealed that CWD in Sekhukhune experienced numerous challenges which affected their social functioning, development and general well-being. Aggravating factors included stigma, labelling and discrimination; disability-specific discrimination and bullying; exclusive education; sexual exploitation; lack of governmental support and poor implementation of disability-specific policies, amongst others.

**Conclusion:**

The provisions of the INDS to promote inclusion, integration, mainstreaming and equitable access to resources and services remained an ideal rather than a reality for CWD in Sekhukhune.

## Introduction

International human rights treaties and African regional human rights frameworks have made provision for human rights protections that apply both directly and indirectly to children with disabilities (CWD). In the South African context, ‘disability’ is defined as:

[*T*]he limitations hindering the full and effective participation of persons with disabilities in society on an equal basis with others, which is expected to last for longer than a year, and which exists after maximum correction or control of the impairment. (National Development Plan 2030 [South Africa] 2015a:7)

Drawing from these international treaties, with the beginning of a democratic government in 1994, South Africa placed the rights of children high on the agenda of all government programmes. These rights are entrenched in Chapter 2 of the Bill of Rights of the South African Constitution (Act 108 of [South Africa] 1996), which stipulates how the needs of every child should be ensured.

However, in practice, many CWD’s lives are characterised by neglect, stigma, discrimination (The African Child Policy Forum [ACPF] 2014; Baffoe 2013) and an institutional failure to implement policies adopted within a developmental and rights-based framework. This is coupled with scarcity of resources across most service sectors in South Africa (Graham 2014:6; Stats SA 2014:9), with the situation in rural communities such as Sekhukhune having become dire. The prevalence of casualties amongst CWD makes it a worthy topic of discussion, not only to illustrate the intensity and complexity of the situation facing CWD, but also to attempt to level the playing field. Better access to basic services begins with examining the challenges they experience, which this study aimed to accomplish in Sekhukhune, Limpopo province, South Africa (United Nations Children’s Fund [UNICEF] 2015:1).

Research on various aspects of disability has been undertaken in various areas of Limpopo province. For instance, Mulugo (2016) investigated factors amongst caregivers that contributed to the development of complications in children living with disabilities in the Vhembe district. Madiba (2015), on the other hand, undertook a study on the experiences of caring for children with intellectual disabilities at the Bana Ba Thari School in the Polokwane municipality of the Limpopo province. From yet another angle, Pilusa (2006) examined the impact of intellectual developmental disorders on family functioning in the Waterberg district of the Limpopo province. We contend that although the above-mentioned studies involved CWD, it was difficult to link their studies with the challenges CWD are confronted with and see this as a gap that needed to be filled.

In South Africa, the White Paper on the Rights of Persons with Disabilities (WPRPD [South Africa] 2015b:146) recommended that any research undertaking on disability must ensure that it advances the rights of persons with disabilities and contributes to the implementation of the Integrated National Disability Strategy (INDS). This study is a direct response to that recommendation. The study aimed to examine the psychosocial challenges of children living with disabilities in Sekhukhune, Limpopo. Based on the identified, articulated and expressed challenges, the study sought to recommend the improvement of the existing INDS for greater responsiveness to the needs of CWD at both the provincial and local levels.

### Conceptualisation of disability

Disability is a complex, dynamic, multidimensional and contested phenomenon. To date, no single definition of disability has achieved international agreement (ACPF 2014:15; World Health Organization [WHO] 2011:3). Various disability organisations, together with researchers from social and health sciences, have identified the role of physical and social barriers in disability. Similarly, Graham (2014:11) described disability as ‘a problem in a body function or structure; an activity limitation, and a difficulty encountered by an individual in executing a task or action’, with restriction of participation in societal activities. This has paved the way for the transition from an individual, medical perspective to a structural, social perspective which perceives people as being disabled as a result of societal conditions (WHO 2011:4).

The United Nations Convention on the Rights of Persons with Disabilities (UNCRPD), as cited by WHO (2011:4), recognises persons with disabilities as individuals living with long-term physical, mental, intellectual or sensory impairments which, when interacting with various barriers, may hinder their full and effective participation in society in comparison with their abled counterparts. This definition provides a basis for understanding disability in relation to the social barriers that restrict full participation over and above impairments, as presented by the social model of disability as supported by ACPF (2014:15).

In this study, disability was conceptualised as a social construct, which entailed an acquired behaviour that can either be reconstructed or deconstructed. This meant that instead of focusing solely on the impairments of CWD, emphasis was placed more on the barriers created by society in limiting their social functioning and full participation in economic and societal activities.

### The prevalence of disability amongst children

According to UNICEF (2013:10), CWD constituted between 93 million and 150 million people globally. However, in South Africa, Statistics South Africa (Stats SA) indicated that about 2 870 130 (7.5%) people in South Africa were living with some form of disability. Of the figures provided, CWD constituted about 25% (718 409) of people living with disabilities (Stats SA 2014:63). It is evident that the number of CWD in South Africa has increased remarkably since the 2005 figure of 5%, to 25% in 2014.

The ACPF (2014:16) stated that the prevalence of disability amongst children was challenging to assess accurately, not only because of difficulties relating to standardised definitions, but because of incomplete data collection and inaccurate statistical results. In agreement, the Department of Social Development (DSD), Department of Women, Children and People with Disabilities (DWCPD) and UNICEF (2012:6) in the South African Situation Analysis report of CWD (2001–2011) posited that measuring disability amongst children is inherently more difficult. This is attributed to the developmental and growth processes which children undergo. This relates to learning to walk, talk, read and write, *inter alia*. These evolving characteristics amongst children complicate the task of assessing functioning and distinguishing significant limitations from variations in normal developmental processes (DSD, DWCPD & UNICEF 2012:6).

Therefore, the researcher enters the caveat that some available figures may be speculative. However, although the available figures may not be an accurate reflection of the prevalence of CWD globally and in South Africa, they are not entirely without significance when considering the magnitude of the challenges facing CWD.

### Factors causing and contributing to impairments amongst children

Globally and in Africa, disability is believed to be caused by a number of factors, which many authors argue could be prevented with proper intervention. Two factors, namely health and poverty are identified and discussed below.

According to Smith (2011:36), preventable disease, congenital malformation, birth-related incidents, psychological dysfunction and physical injury all constituted child disability. In its African report on CWD, the ACPF (2014:19) stated that in Africa, illness-related infection accounted for about 65% of disability cases amongst children, complications during birth and birth processes accounted for 17%, whilst accidents accounted for 11%. Over the past decade, human immunodeficiency virus (HIV) and acquired immunodeficiency syndrome (AIDS) had also contributed significantly to impairments amongst children globally (Njelesani 2013:13).

Poverty and disability were inextricably connected in that disability was both a cause and a consequence of poverty (UNICEF 2013:16; WPRPD 2015b:22). The Innocenti Research Centre (IRC) (2007:5) maintained that the correlates of poverty, such as inadequate medical care and unsafe environments, significantly contributed to the incidence and impact of childhood disability and further complicated efforts for prevention and response.

The ACPF (2011:14) posited that poverty was experienced mostly in underdeveloped communities and that children living in rural areas were disproportionately more susceptible to being born disabled than their counterparts in urban areas. Considering the inextricable link between poverty and disability, IRC (2007:6) argued that effective action to reduce poverty would address and reduce the prevalence of disability amongst children.

### Psychosocial challenges experienced by children with disabilities

The psychosocial concept denotes interaction of environmental circumstances on the minds of individuals. It refers to any aspect connected to the psyche, consciousness, personality and social context of an individual (Kirst-Ashman 2010:176). In this study, psychosocial referred to influences of social factors on the psychological and behavioural components of CWD.

Children living with disabilities need care and support to enable them to cope and function effectively. The United Nations Children’s Fund (2013:10) asserted that even though provision had been made through legislative frameworks to promote and protect the rights of CWD, social barriers continued to restrict their effective participation. Baffoe (2013:187) and World Health Organisation (WHO) (2011:6) agree with researchers’ views in this study that these societal barriers limited CWD’s ability to access opportunities, privileges and resources and to participate fully in the life of their community. Some of the stated barriers included ‘social exclusion, stigma, discrimination and prejudice, negative societal perceptions, ritualistic killings of CWD and exclusive education system’.

Central to the many problems faced by CWD is social exclusion. It is defined by Giddens and Sutton (2017:543) as ways ‘in which individuals may become cut-off from full involvement in the wider society’. It entails social factors that prevent CWD from accessing equal and equitable opportunities presented to most of the population. The problem of disability lies not only in the impairment of function and its effects on individuals, but more importantly, in their relationship with broader society. The marginalisation of CWD infringes on their democratic rights and makes them vulnerable to abuse and ill-treatment (ACPF 2014:3; Patel 2005:175).

Negative attitudes towards CWD are pervasive in different spheres of society. These attitudes were evident from high-level authorities and policy enactors in government to traditional and religious leaders in urban and rural communities. They paved the way for the marginalisation of people living with disabilities and deprived them from partaking in socio-economic activities. (ACPF 2014:29). Adding to the struggles faced by CWD was stigmatisation from family members, fellow learners, friends and the community at large, making them feel inferior and unaccepted. Supporting this view, Giddens (2006:269) explained that stigma was a relationship of devaluation in which an individual was disqualified and rejected by society. Further related to stigma is discrimination, which denotes unfair treatment and prejudice based on societal preconceived ideas without substantiated facts, thus devaluing a person’s self-esteem, worth and dignity (Patel 2005:303).

Negative beliefs about what causes disability and the limitations of people with disabilities were often firmly held by societies and were difficult to dispel. Such societal perceptions and attitudes were mostly based on misconceptions, fears and misunderstandings that exposed CWD to prejudice and discrimination, giving rise to the denial of their basic human rights and access to the societal resources accorded to their abled counterparts (Baffoe 2013:188). Exacerbating the challenges faced by CWD was the negative treatment by their family members, which included name-calling, labelling and restrictions from performing certain household chores (ACPF 2014:32 & 34; Njelesani 2013:25).

A disturbing outcome of negative attitudes was the brutal and ritualistic killings of CWD because of superstitious and erroneous beliefs. In some cases in Africa, CWD were subjected to abuse and some were killed for body parts. In February 2018 in Mpumalanga, South Africa, two children, one of whom was living with albinism, were kidnapped and killed for their body parts (The Citizen 2018). The United Nations (UN) reported that in some communities, erroneous beliefs and myths, heavily influenced by superstition, put the security and lives of CWD at constant risk. These beliefs and myths were centuries old and were present in cultural attitudes and practices around the world (UN 2013). An understanding of these differences and complex interactions was fundamental in addressing disability-related stigma and discrimination within communities (ACPF 2014:31–32).

Most CWD remain excluded from equal access to education and its associated benefits. This has a detrimental implication in contending for better jobs in the future and making positive marks within the economic spectrum, with most CWD in rural areas being at risk (UNICEF 2013:20). According to the IRC (2007:17), CWD’s right to education is enshrined in human rights treaties, including articles 28 and 29 of the United Nations Convention on the Rights of the Child (UNCRC). A critical implication is the requirement for education to be compulsory and to be made freely available for all including CWD, and if necessary, for the state to provide financial assistance (Tesemma 2011:8).

### Disability legislative frameworks

Since the inception of democracy in 1994, South Africa has made some progress in ensuring the protection of all its citizens, including CWD. Through the Constitution and the Bill of Rights, the country has enacted and ratified an enabling legislative framework based on the values of freedom and equality for all. This takes into cognisance efforts to recognise the rights of CWD at both the national and international levels and to mainstream disability into the development agenda (ACPF 2011:17; IRC 2007:32; Stats SA 2011:7). The following policies were central to the study:

- The White Paper on Disability was implemented to update the 1997 INDS, to integrate obligations in the UNCRPD and to respond to the Continental Plan of Action for the African Decade of Persons with Disabilities (1999) as asserted by ACPF (2011:18). Its central objective was premised on the protection and promotion of the rights of people living with disabilities in South Africa (WPRPD 2015b:38). Specifically, the overall purpose of the WPRPD is to provide a mainstreaming trajectory for realising the rights of people with disabilities, including CWD, through the development of targeted interventions that seek to remove barriers and apply the principles of universal design. It guides the assessment of all existing policies and ensures that the development of new sectoral legislations and policies, programmes and budget and reporting systems are in line with the Constitution and the ratified international treaties for the protection of the rights of CWD (WPRPD 2015b:38).
- The Integrated National Strategy (INS) policy document supplements the provisions made in the 1997 INDS document by categorically focusing on addressing the needs of CWD within the South African context. As a guiding legislative framework for CWD, the INS incorporates the recommendations and provisions of ratified and adopted international and regional treaties, as well as local policies protecting and promoting the rights of CWD. The principles of the INS include respecting the dignity and the right to survival for CWD, realisation of fundamental freedom and active participation in the community, promotion of self-representation and participation in the decision-making process, access to basic education and developmental opportunities accorded to other children and equitable access to resources (South Africa 2009:26).

### Theoretical framework guiding the study

This denotes the set of transportable ideas used to formulate, predict and understand the phenomenon of a research study (Rubin & Babbie 2005:43; Shaw et al. 2013:282). The theories outlined below gave direction to the study.

This study was based on the ecological systems perspective developed by Bronfenbrenner. Gitterman and Germain (2008:2) explained that the ecological thinking model focuses on the reciprocity between people and their environment. According to Teater (2014:25), the fundamentals of an ecological perspective are based on the person-in-environment spectrum which denotes the transactional fit between CWD and their environments. The key elements of the ecological perspective are the growth, development and potentialities of CWD, taking into account the attributes of their environment that support or undermine the realisation and expression of their potential.

**FIGURE 1:**
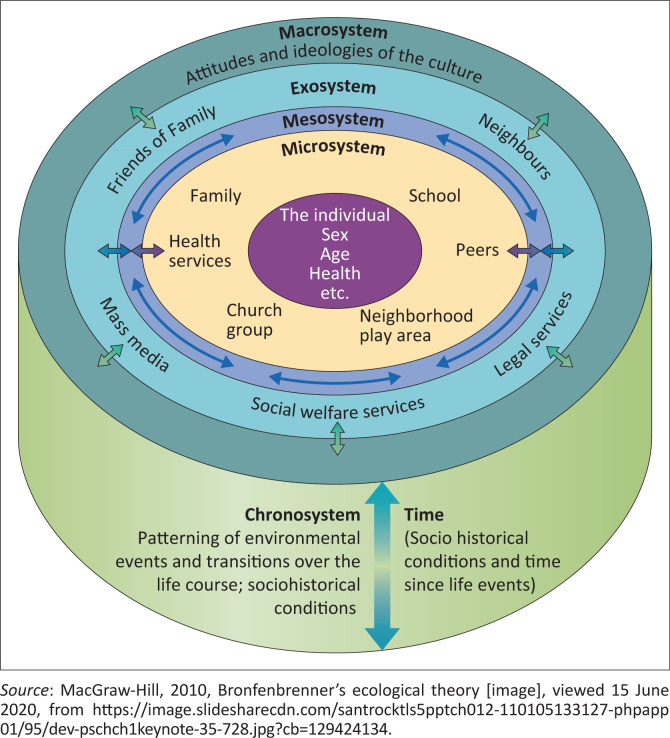
Bronfenbrenner’s ecological systems perspective.

The rationale behind the application of the ecological systems perspective was to develop an understanding of the adaptive fit or lack thereof between CWD and their environments. Figure 1 provides an illustration of the transactional fit between CWD and different systems and subsystems impacting on their psychosocial functioning.

## Research methodology

Research methodology constitutes the blueprint of scientific inquiry and provides a means through which intellectual development and understanding of phenomena are executed and enhanced (May 2011:1; Taylor, Bogdan & DeVault 2015:14; Walliman 2016:24). The following research methodology was employed.

### Research approach

The study employed an interpretivist approach within a qualitative mode of inquiry. Hennink, Hutter and Bailey (2011:8) explained that qualitative research permits the researcher to examine people’s experiences in detail by using specific sets of research methodologies. It seeks to study a research problem from a local perspective as presented by the research participants (Jha 2014:102). On the other hand, the interpretivist paradigm typically involves participants’ own written or spoken words and observable behaviour. It is concerned with developing explanations of social phenomena as detailed by the research participants (Hancock, Ockleford & Windridge 2009:7).

This approach was oriented towards exploring the phenomenon of child disability intensively to provide a detailed description of the psychosocial challenges experienced by CWD in Sekhukhune. This type of research paradigm typically studies participants or systems by interacting with and observing them in their natural environments and focusing on their meanings and interpretations (Moriarty 2011:2). In this study, interacting with CWD and the people who render services to them and locating them from their schools, homes and community was imperative.

### Research design

Research design constitutes the blueprint for the collection, measurement and analysis of data and subsequently indicates which methods are appropriate for the study (Allmer 2012:1; Sahu & Kumar 2013:25; Walliman 2016:37). Based on the qualitative components of the study, phenomenology and descriptive research designs were employed. Rubin and Babbie (2016:304) asserted that phenomenology entailed the philosophical paradigm for conducting qualitative research that emphasised people’s subjective experiences and interpretations of the world. It was founded on the ideology that rich information is unearthed from participants’ constructs and sense of their lived experiences, which is in line with a qualitative approach.

In the context of the study, this meant eliciting rich data regarding the psychosocial challenges experienced by CWD as narrated first-hand. On the other hand, descriptive research design was concerned with providing rich details about the participants’ environment, interactions, meanings and everyday lives, thus providing a broad description of the phenomenon as detailed by the research participants (Rubin & Babbie 2016:61; Walliman 2011:10).

### Target population

The target population for the study was CWD, both male and female, residing in Sekhukhune within the age range of 10–17 years. They are children as defined in the *Children’s Act 38* of 2005 (South Africa 2005), 2014 (amended); ‘child’ means a person under the age of 18 years. In this study, a child is defined as falling within the ages of 10 and 17 years as they were presumed to be in school and using services such as transport, health care services and other community facilities. As such, they were considered able to share their experiences first-hand.

An in-depth individual interview was conducted with each in their home setting under parental supervision. The interview took between 50 min and 60 min using a semi-structured interview schedule. Interviews were conducted in Sepedi to accommodate those participants who did not understand English and only comprised of seven from a sample of ten. The study population also included key informants who worked in close collaboration with CWD and were knowledgeable about the challenges CWD in Sekhukhune experienced. Key informants included schoolteachers from both mainstream and special schools, health care practitioners, community leaders and social workers who worked in close collaboration and provided diverse services to CWD. Also forming part of the key informant group were parents and/or primary caregivers of CWD who fulfilled caring and rearing roles, and who were well suited to providing rich descriptions of the challenges encountered by CWD. These were people who frequently interacted with CWD and had information regarding the psychosocial challenges impairing their social functioning and general wellbeing within Sekhukhune. The said institutions were approached and permission requested from the education department and DSD for children in special schools. The Department of Health also gave permission for the three health care professionals. These were key informants (36; 10 parents, four caregivers, three social workers, three teachers, three health practitioners and three community leaders) who were subjected to a focus group with a set of questions that allowed for in-depth discussions. The remaining three CWD also participated in a focus group in the special school where they resided. These are children whose disabilities included blindness and albinism. This was held in a private room free from disruptions.

### Sampling technique and sampling type

The study employed a non-probability sampling technique which bases its notion on the assertion that the odds of selecting any participants in the study are not known. The sampling process was in line with the qualitative approach, phenomenology and descriptive research designs guiding the study (eds. De Vos et al. 2011:394; Rubin & Babbie 2016:61). The study employed two types of non-probability sampling strategies, viz. purposive and snowball sampling. Ritchie et al. (2013:113) stated that in purposive sampling, the selection of participants, setting or other sampling units is criterion-based, which was a sample of CWD in the age range of 10–17 years. Children from both genders (that is, male and female) were selected who were positioned to reason logically and to provide first-hand information. Informed consent was sought from their parents and caregivers. The snowball and/or chain sampling was used to locate one or two families of CWD, and then asked them to name other likely participants. This form of sampling facilitated the identification of hard-to-find participants as asserted by Babbie and Mouton (2001:167). This was consistent with the qualitative approach and phenomenology design guiding the study (Creswell 2016:109; Walliman 2016:115).

### Unit of analysis (sample size)

The sample was composed of 36 participants who were purposefully selected, comprising 10 CWD, 10 parents, four caregivers, three teachers, three social workers, three health care practitioners and three community leaders who worked in close collaboration with CWD in Sekhukhune. The final sample size was adjusted, however, based on the principle of theoretical saturation, in which the researcher stopped interviewing once it was found that participants were repeating similar information, with nothing new forthcoming (Maree 2011:82).

### Data collection

The essence of the qualitative data collection process is to capture perceptions and lived experiences as narrated by participants, translating them into rich analyses and building credible findings relating to the study phenomenon (Kabir 2016:202). Data were collected using three triangulated methods, viz. in-depth individual interviews (seven of the 10 CWD), focus group discussions and key informants’ interviews (parents, teachers, caregivers, social workers, community leaders and three CWD). Honorene (2017:91) asserted that triangulation is a powerful technique which promoted validation of data through cross-verification. All sessions were conducted once and lasted between 50 min and 60 min.

### Data analysis

In this study, thematic data analysis was employed. This refers to the process of identifying, analysing and reporting patterns or themes in data as postulated by Whittaker (2012:96). The rationale behind selecting thematic analysis was that the crux of qualitative analysis is to bring meaning to respondents’ words by identifying themes or patterns, ideas, concepts and/or behaviours that will facilitate understanding and then organising them into coherent categories that summarise and bring meaning to the text (Maree 2011:110). The steps followed in thematic analysis was guided by Bricki and Green (2007:23–24), which involved the following: reading and annotating transcripts, identifying themes, developing coding scheme, coding data and producing the final report.

### Ethical considerations

This refers to the preferences that determine and influence the behaviour and conduct of researchers when conducting a study with experimental subjects or human participants (eds. De Vos et al. 2011:114). Ethical approval was sought before the study could commence and from entities like Department of Social Development (DSD), hospitals and gatekeepers. Ethical approval was acquired from North-West University’s Research Ethics Regulatory Committee (reference number NWU-HS-2017-0191) and from the Limpopo Provincial Research Ethics Committee (reference number REC-111513-038). This process was completed with numerous additions and deletions as recommended by the review committee. When this process was concluded to the satisfaction of the ethics committee, Limpopo provincial research ethics committee was approached and permission was granted, whereafter data collection ensued.

To ensure that the research is ethically sound, and that no harm was inflicted on participants, the following key ethical guidelines were applied which included informed consent, voluntary participation, privacy, confidentiality, anonymity, beneficence and non-maleficence (Bless, Higson-Smith & Kagee 2006:143; eds. De Vos et al. 2011:116–117).

## Results

This section presents and discusses the findings of the study, synthesised with direct quotes from participants, the literature and findings of various researchers within the disability context. Given the findings of the study, the following themes outlining the psychosocial challenges experienced by CWD in Sekhukhune emerged.

### Theme 1: Stigma, labelling and discrimination

Information gathered showed that some children at school and within their homes did not want to associate, interact with or play with CWD because of their disabilities. Considering the findings of the study, it is evident that such discriminatory practices were perpetuated by a lack of knowledge by some educators as well as peers on disability-related issues and on ways to accept and live with CWD. In other instances, CWD were labelled by their peers, family members and community members in relation to their specific disabilities, whilst others imitated their impairment, which has an adverse impact on CWD’s confidence, self-efficacy and self-esteem. Without proper intervention, this can perpetuate isolation and withdrawal from societal activities.

A participant from a mainstream school stated that:

‘[…*W*]hen I walk around the school during break-time, some learners will imitate the way I walk and laugh at me and others would call me names….’ (Participant 4, Learner with disability)

A social worker commented:

‘Discriminatory attitude has a lot of bearing on their functioning, especially on their psychological functioning because they do not see themselves as human beings. Secondly, they start comparing themselves to others as ways of understanding what distinguishes them from others. And they also have… [*pause*] they start to develop withdrawal on some activities. They start feeling inferior and looking down on themselves. They don’t believe in their capabilities.’ (Participant 3, Social Worker)

Confirming these findings, various authors within the context of disability have argued that a lack of knowledge and awareness of disability and their associated characteristics perpetuated the adverse way CWD were received and treated by society (ACPF 2014:29; Rohwerder 2018:2). In addition, Baffoe (2013:193) asserted that, apart from the personal pain and moral degradation brought on by stigma, labelling and discrimination, such behaviours could also prevent CWD from seeking help or participating in societal activities and could see them withdrawing altogether.

### Theme 2: Disability-specific discrimination and bullying

From the caregivers, it was found out that children bully each other too. This occurred where a differently disabled child would bully another with a different disability, for example, deaf children bullying and discriminating against blind children. This behaviour tended to promote disability subcultures within the school, that is, deaf children grouping themselves as a unit, and likewise with blind children and children with albinism.

A participant from the caregivers had this to say:

‘Children from the deaf section don’t get along with children from the blind section. They always bully blind children and steal their food, knowing that they cannot chase or identify them….’ (Participant 5, Caregiver)

Another participant confirmed that:

‘[…*I*]t has become the norm that one has to associate and become friends with children who share a similar impairment. In that way it makes it easy for us to interact.’ (Participant 7, Learner with a disability)

Lipson and Rogers (2000:213) stressed that CWD sharing a subculture usually shared a language or communication pattern, values and interaction patterns which distinguished them from others. Children with disabilities forming part of subculture groups tended to share values and the lifestyle of the dominant disability culture, but also endeavoured to maintain their distinctive mores and lifestyles which, without proper guidance, could promote discrimination and other harmful practices related to bullying and marginalisation of CWD who were not within their defined subculture.

The conclusion that could be drawn from these findings is that such discriminatory practices perpetuated segregation amongst CWD based on the belief that certain disabilities were superior to others, thus promoting isolation and marginalisation. Ervin (2011:8) agreed that bullying had an adverse effect on the child experiencing the ordeal, which often led to withdrawal, anxiety, depression and suicidal thoughts. Furthermore, bullying interfered with CWD’s learning and performance at school, which perpetuated poor results and the risk of dropping out.

### Theme 3: Lack of special schools and exclusive education (mainstreaming)

The study found that the lack of special schools was a challenge for most CWD in Sekhukhune. The available schools were unable to cater for the majority of CWD in the district, and children were compelled to travel to other districts or provinces in search of schools tailored for their specific disability.

A participant explained:

‘My family tried to find a special school closer to my home that caters for deaf children but without luck, and as such I had to come and study at the Special School for the Deaf and Blind, because there are no schools in my village that teach braille….’ (Participant 7, Learner with a disability)

A social worker reported:

‘The system is unable to accommodate these children, and in some instances, you find teachers saying that they cannot work with a disabled child, and they ask if we (social workers) can’t find another school for them…[*pause*] and the special schools which I know are few, very, very, few. So that is why most children don’t attend school, they just stay at home….’ (Participant 3, Social Worker)

Confirming this finding, the Integrated National Strategy on Support Services to CWD (South Africa 2009:10) posited that the education system had failed to promote access to education for CWD. This was attributed to the finding that mainstream schools were not yet equipped to accommodate CWD (Education White Paper 6 [South Africa] 2001:9), and teachers had not been trained to integrate disability-specific teaching and learning into their curriculum, thus leaving CWD excluded from mainstream education, as confirmed by UNICEF (2013:20). Given the findings, it is evident that much still needs to be done to ensure that CWD were fully mainstreamed and for the principle of inclusive education to be realised in Sekhukhune. Learners with cerebral palsy or autistic learners and those with other disabilities of a physical nature may not attend a mainstream school and would normally be referred to ‘special schools’, hence the exclusion. It is also a concern that schools are not readily prepared to accommodate CWD, regardless of the type of the disability, and that no efforts have been made to equip teachers on how to handle such children, thus reducing the stigma which in itself is a challenge to CWD. The Integrated National Strategy is meant to work collaboratively with all stakeholders and so make sure that inclusivity is upheld.

### Theme 4: Neglect

The findings revealed that some CWD in Sekhukhune were neglected by their parents and caregivers. A concern was raised regarding CWD attending special schools who stayed in the schools’ boarding facilities and who had been neglected by their families, who had not ensured that they had all the items they needed to meet their specific needs. It was found that CWD were taken to school with incomplete uniforms, on many occasions dirty and ragged.

On this point, a social worker stated:

‘The most frequently reported cases are for neglect. You find that these children don’t have clothes, don’t have snacks and they don’t have shoes. These are some of the cases that are reported. They don’t care if the child stays at boarding… [*pause*] they just let them leave without anything. For those who are blind, they don’t even check if the child has enough clean clothes or not…is like they take advantage that the child cannot see.’ (Participant 1, Social Worker)

Furthermore, medical neglect which entailed depriving CWD of access to medical care emerged in the findings. Health care practitioners disclosed that some families did not adhere to regular medical procedures by taking CWD to health care facilities for their regular medical assessments, check-ups and/or immunisations, thus putting their health at risk. Educators are not always equipped to deal with learners with disabilities and as such cannot intervene on their own, unless parents have briefed them on the learner’s disability. The school will intervene if it is an emergency by, for instance, calling an ambulance should such an incident occur.

A nurse stated:

‘At times, parents don’t bring children to clinic for growth monitoring. They tend to hide these children at home and bring them to the clinic when they are really sick and unable to do anything. That is when they are brought for medical treatment.’ (Participant 1, Nurse)

The ACPF (2014:29) maintained that these negative attitudes paved the way for the deprivation and marginalisation of CWD in societal activities and access to services. A child with disability alluded to the far-flung resources as a barrier, explaining thus: ‘…there no facilities here, even the South African Social Security Agency (SASSA) office is in Mogaung village. We have to take a taxi to Luckau or Groblersdal to access them’ (Participant 2).

### Theme 5: Sexual exploitation

Key informants reported that CWD in Sekhukhune were subjected to various forms of abuse and maltreatment at home and within the community. The recurring abuse recounted by key informants was sexual exploitation, whereby community members took advantage of these children’s vulnerabilities.

A social worker reported:

‘Children with hearing impairment [*deaf*] are mostly abused sexually. People take advantage just because they cannot communicate verbally and also because they are unable to relate their story to others.’ (Participant 1, Social Worker)

### Theme 6: Poor infrastructural development and lack of facilities

Lack of infrastructural development impeded effective integration, accessibility and accommodation for CWD in Sekhukhune. The village had no proper roads to accommodate CWD in wheelchairs and no recreational facilities for CWD. The findings indicated that in most development programmes, disability-specific programmes had been overlooked. For instance, no sports facilities had been specially developed to accommodate CWD, and the available facilities did not meet the prescribed standards and posed a risk to the functioning, development and general well-being of CWD.

Confirming this, a social worker said:

‘We have one stimulation centre and the service they provide is poor, extremely poor, and the hygiene is a challenge. We have a multidisciplinary team from health, occupational therapists and so forth visiting the centre but nothing is done to improve the condition of that centre, and to my surprise the government still funds such facilities which pose a threat to these children.’ (Participant 3, Social Worker)

Agarwal and Steele (2016:3) concurred that accessible infrastructure promoted an inclusive environment for CWD and allowed them to enjoy their civil, social and economic rights. It also encouraged independent living and equal participation in societal activities. Conversely, an inaccessible environment with an underdeveloped infrastructure promoted exclusion from education, social contacts and full integration into the life of the community.

### Theme 7: Shortage of health care facilities

The shortage of health care facilities in various municipalities within Sekhukhune compounded the psychosocial challenges faced by CWD. Most villages had no health care facilities, compelling CWD and their parents or caregivers to travel long distances or to go to neighbouring villages to access health care facilities.

A social worker commented:

‘Fetakgomo itself does not have a hospital, so the children have to be transferred to another municipality just to get assistance, and it does not make sense. The clinics we have only work for a few hours; some work till 8:00 PM or 9:00 PM, so when children have problems during the night, where are we supposed to take them to because we don’t even have a hospital? … It all becomes a burden and a strain for children to access a health care facility and be assisted.’ (Participant 3, Social Worker)

The lack of appropriate or accessible health care services was argued to have an adverse impact on CWD. Participants maintained that, based on the issue of inaccessibility, some CWD rely on self-diagnosed or over-the-counter medication, and some do not adhere to regular immunisation.

On this note a social worker posited that:

‘It impacts on the children with disabilities negatively as some of them will resort to self-diagnosed medication, which is not good for them as they need to be assessed by a medical practitioner who will make a prescription for them. Some might not even go for their appointments and for immunisation, you see? … [*pause*] so there are a lot of implications.’ (Participant 3, Social Worker)

Section 27 of the Constitution and article 23 of the **United Nations Convention of the Rights of the Child (UNCRC)** promote the right to health care, stipulating that CWD, like all other citizens, had the right to access basic health care services, including the right to reproductive health care, and that these provisions had to be made freely available within their area of residence, which was not always the case for CWD in Sekhukhune (ACPF 2014:43; Constitution of the Republic of South Africa 1996:11; UN Human Rights 1989:7).

### Theme 8: Lack of government support

The findings revealed that a lack of government support in enhancing the functioning of CWD in Sekhukhune impeded the integration of CWD into mainstream society. The United Nations Children’s Fund (2013:4) emphasised that under the UNCRPD, CWD and their families had the right to an adequate standard of living and were entitled to subsidised or free support services such as free education, day care, respite care and social protection. Based on the findings of this study, such provision remained an unrealised ideal for CWD in Sekhukhune.

A parent commented:

‘Government promised us that child with disability will attend school for free but these children are still made to pay school fees. My concern is that does free education cater only for abled children, because these ones (children with disabilities) are discriminated against. We have to pay R1500.00 for their fees and yet we are told that education is free.’ (Participant 1, Parent)

The ACPF (2011:5) maintained that the government should exempt children from paying school fees as a way of enhancing the right to education and increasing enrolment and retention of children who would otherwise have dropped out.

### Theme 9: Poor implementation of disability-specific policies

The study’s findings showed that poor implementation and monitoring of disability-specific policies to promote integration, mainstreaming, accessibility and inclusiveness of CWD in Sekhukhune remain an obstacle.

A parent maintained that:

‘Our policies protect the rights of children with disabilities. The Constitution has made the provision on the protection of people living with disabilities and is against any form of discrimination, but the issue is that such policies are being overlooked and poorly implemented.’ (Participant 1, Parent)

Judy et al. (2014:55) argued that although there has been expansion in inclusive mainstream education policies, the evidence remains skewed and unclear on the effectiveness of these developments in augmenting the level of inclusiveness for CWD. It was found that a lack of training regarding a disability-specific framework and policies for service providers hampered the effective and efficient rendering of services to CWD.

In this regard, a caregiver maintained that:

‘They must capacitate us on frameworks and guidelines that focus on children with disabilities. They must provide us with workshops so we can be informed about these children.’ (Participant 2, Caregiver)

The researchers are of the view that not all schools or district offices have really put an effort in implementing inclusive education. The gap is seen in the school heads as they do receive documents, reviews and some policies and should make a point that all involved parties are vested with the information. As already mentioned, children with different disabilities cannot be put under one umbrella when it comes to excluding them from mainstream education. Blind learners come to mind here; they have a special writing system and the schools are not disability-friendly, lacking wheelchair ramps, guide-dogs and other assistive devices that the learner with a disability would need.

## Summary of findings

An assortment of challenges faced by these children in the rural areas of Sekhukhune was explored in this study. The study revealed that CWD were subjected to many psychosocial challenges, found to be detrimental to their social functioning, development, and general well-being. The findings of different authors revealed, as shown in the reviewed literature, the challenges experienced by CWD in different countries and in South Africa, most of which were confirmed in this study. The challenges faced by CWD included exclusive education, stigma, labelling, discrimination, poor infrastructural development, neglect, sexual exploitation, *inter alia*. The findings suggest that the ineffectiveness of the Integrated National Strategy (INS) in addressing the psychosocial challenges experienced by CWD in Sekhukhune could be attributed to a lack of government support and poor implementation of disability-specific policies. Addressing this issue would require concerted, integrated and coordinated effort from various spheres of government to ensure that the provisions made in the INDS and other legislative frameworks promoting and protecting the rights of CWD on an equal basis with others are ensured. The first step is to convene workshops and information sessions to share the findings with relevant stakeholders. The relevant stakeholders would be the Director General, who would give directives to directors reporting to him. It is hoped that from this angle especially, the Sekhukhune district in particular would initiate the recommendation after the findings have been shared and start with the process; the other provinces can follow suit, so as to reach out to all CWD. The aim is to ensure that all are informed and obtain their buy-in. The paper has been presented to the conference that took place in 2021. The findings further outlined the need to ensure that education is made freely accessible to CWD.

### Recommendations

Given the findings of the study, the overarching recommendation is to address the challenges faced by CWD in Sekhukhune by promoting the responsiveness of the integrated disability strategy.

Flowing from the above-named recommendation, the following recommendations are proposed:

1. That the provisions made in various international and local declarations and policies are fully implemented. This includes training of professionals on disability-related frameworks and providing the necessary resources toward the realisation of the provisions made in disability-specific legislations.
2. That government put measures in place that prioritise the care, protection and integration of CWD in terms of resources to ensure that these children are supported.
3. To ensure that the provisions of the Integrated National Strategy in support services to CWD are realised, including addressing gaps and barriers in service accessibility, alleviating poverty, increasing measures for social grant accessibility and improving service delivery in rural areas.
4. To develop monitoring and evaluation mechanisms to assess progress in the implementation of the provisions of the Integrated National Strategy in the promotion of inclusiveness, integration, accessibility, mainstreaming and eradication of poverty amongst this vulnerable group in rural and under-developed areas.
5. This can, therefore, be attained by promoting access to inclusive education for CWD. The government should provide a full subsidy to cover the fees and other education-related expenses such as transport costs, boarding or hostel fees and learning materials.

## Conclusion

This study has proven that CWD in Sekhukhune face many challenges which may not only be physical, but psychosocial as well. It has been found that CWD are discriminated against, that they are being bullied and stigmatised. Some are neglected, sexually exploited and many more experience shortage of health care, lack of appropriate infrastructure and exclusion from mainstream education. It is hoped that, with the recommendations put forth, more care and support will be forthcoming as directed in the INDS. The plight of CWD has been shared with interest groups and papers were presented in line with some of the interventions and awareness-raising in support of the integrated strategy.
